# Does Bypass of the Proximal Small Intestine Impact Food Intake, Preference, and Taste Function in Humans? An Experimental Medicine Study Using the Duodenal-Jejunal Bypass Liner

**DOI:** 10.3390/nu14102141

**Published:** 2022-05-20

**Authors:** Madhawi M. Aldhwayan, Werd Al-Najim, Aruchuna Ruban, Michael Alan Glaysher, Brett Johnson, Navpreet Chhina, Georgios K. Dimitriadis, Christina Gabriele Prechtl, Nicholas A. Johnson, James Patrick Byrne, Anthony Peter Goldstone, Julian P. Teare, Carel W. Le Roux, Alexander Dimitri Miras

**Affiliations:** 1 Community Health Sciences Department, College of Applied Medical Sciences, King Saud University, Riyadh 11451, Saudi Arabia; maldhwayan@ksu.edu.sa; 2 Diabetes Complications Research Centre, Conway Institute, School of Medicine, University College Dublin, D04 V1W8 Dublin, Ireland; carel.leroux@ucd.ie; 3 Department of Surgery and Cancer, Imperial College London, London SW7 2BX, UK; aruchuna.ruban@nhs.net (A.R.); j.teare@imperial.ac.uk (J.P.T.); 4 Department of General Surgery, University Hospital Southampton NHS Foundation Trust, Southampton SO17 1BJ, UK; michaelglaysher@me.com (M.A.G.); james.byrne@uhs.nhs.uk (J.P.B.); 5 Department of Metabolism, Digestion and Reproduction, Imperial College London, London SW7 2BX, UK; brett.johnson@nhs.net (B.J.); a.miras@nhs.net (A.D.M.); 6 PsychoNeuroEndocrinology Research Group, Centre for Neuropsychopharmacology, Division of Psychiatry, Department of Brain Sciences, Imperial College London, Hammersmith Hospital, London SW7 2BX, UK; navchhina@gmail.com (N.C.); tony.goldstone@imperial.ac.uk (A.P.G.); 7 Department of Endocrinology, King’s College Hospital NHS Trust, London SW7 2BX, UK; georgios.dimitriadis@kcl.ac.uk; 8 Imperial Clinical Trials Unit, Department of Public Health, Imperial College London, London SW7 2BX, UK; c.prechtl@imperial.ac.uk (C.G.P.); nicholas.johnson@imperial.ac.uk (N.A.J.); 9 Centre for Diabetes, Ulster University, BT52 1SA Coleraine, Ireland

**Keywords:** Endobarrier, obesity, food preferences, eating behaviour, taste function

## Abstract

The duodenal-jejunal bypass liner (Endobarrier) is an endoscopic treatment for obesity and type 2 diabetes mellitus (T2DM). It creates exclusion of the proximal small intestine similar to that after Roux-en-Y Gastric Bypass (RYGB) surgery. The objective of this study was to employ a reductionist approach to determine whether bypass of the proximal intestine is the component conferring the effects of RYGB on food intake and sweet taste preference using the Endobarrier as a research tool. A nested mechanistic study within a large randomised controlled trial compared the impact of lifestyle modification with vs. without Endobarrier insertion in patients with obesity and T2DM. Forty-seven participants were randomised and assessed at several timepoints using direct and indirect assessments of food intake, food preference and taste function. Patients within the Endobarrier group lost numerically more weight compared to the control group. Using food diaries, our results demonstrated similar reductions of food intake in both groups. There were no significant differences in food preference and sensory, appetitive reward, or consummatory reward domain of sweet taste function between groups or changes within groups. In conclusion, the superior weight loss seen in patients with obesity and T2DM who underwent the Endobarrier insertion was not due to a reduction in energy intake or change in food preferences.

## 1. Introduction

The impressive weight loss observed after RYGB surgery is caused predominantly through a reduction in appetite and hence food intake [1,2]. However, a subgroup of patients also change other aspects of their eating behaviour, including food preference [1]. This shift away from energy-dense sweet and/or fatty foods to less energy-dense options is thought to be an additional mechanism underlying weight loss [3,4]. The gap in our current knowledge is which component of the RYGB gut manipulations underlies the observed changes in dietary behaviour.

The manipulations with RYGB include the formation of a small gastric pouch which is anastomosed to the proximal jejunum, bypass of the stomach and proximal small intestine through which the biliopancreatic secretions still flow and mix with food at the jejuno-jejunal anastomosis and throughout the common channel.

Animal models of the duodenal-jejunal bypass operation have contributed to our understanding of the role of proximal intestinal bypass on eating behaviour. Mice that underwent duodenal-jejunal bypass (DJB) surgery exhibited lower sugar intake in a sweet-seeking task compared to sham-operated mice [5]. The mechanism was thought to involve disrupted gut-brain signalling in the DJB mice, in which duodenal glucose infusions caused a higher release of dopamine than jejunal glucose infusions in the dorsal striatum of sham mice. This effect was significantly diminished in DJB mice [5]. This observation leads to the hypothesis that bypass of the proximal small intestine might be the component of the RYGB manipulations responsible for the reduction in the preference for sweet/fatty foods after surgery.

We adopted a reductionist approach and used the duodenal-jejunal bypass liner (Endobarrier device, GI Dynamics, Lexington, MA, USA) as a research tool to enable us to address our hypothesis in humans. The Endobarrier is a 60 cm fluoropolymer sheath that is inserted endoscopically, anchored at the duodenal bulb and lines 60 cm of the proximal small intestine. We previously demonstrated in the largest RCT in the field that the Endobarrier causes superior weight loss to lifestyle modification in people with obesity and T2DM [6].

The aim of this experimental medicine study was to determine the impact of the Endobarrier device on food intake, food preferences and taste function in humans.

## 2. Materials and Methods

### 2.1. Patients and Study Design

**Figure 1:**
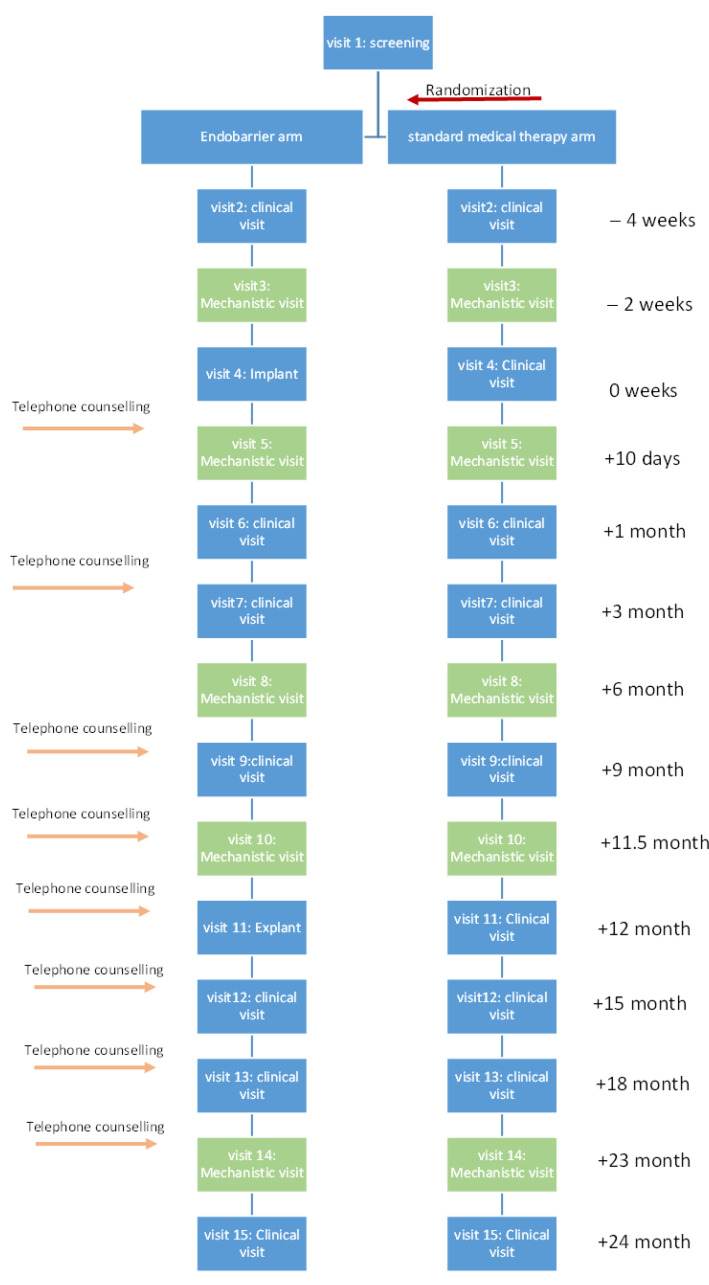
Trial design.

This was a nested mechanistic study within a larger randomised controlled trial comparing the impact of lifestyle modification with vs. without Endobarrier insertion in patients with obesity and T2DM [6]. The study took place in two academic centres, investigational sites—Imperial College London and University of Southampton. Patients were recruited and followed up in the NIHR Imperial and Southampton Clinical Research Facilities. A complete description of the trial protocol was previously published [7]. In brief, the trial was conducted over 2 years (1 year of treatment and 1 year follow up), 160 participants were randomized at a 1:1 ratio to one of the two study arms. For this nested study, data were collected at 5 time points (mechanistic visits): at baseline (2 weeks before intervention), 10 days, 6 months, 12 months, and 24 months post-intervention (Figure 1).

The Endobarrier is an impermeable fluoropolymer sleeve inserted endoscopically through the duodenum and into the jejunum. The sleeve is open at both ends allowing for chyme passage from the stomach into the lower jejunum, bypassing nutrient absorption along its length by creating a barrier between the partially digested food and the absorptive surface of the small intestine [8]. Implanting the device takes an average of 45 min, and the implant is performed under general anaesthetic. The device barbs are anchored to the duodenal bulb 5–10 mm away from the pylorus. The sleeve then extends for 60 cm through the duodenum by peristalsis movement. Device explant is also done under general anaesthesia, taking, on average, 30 min to perform. The participant is usually discharged to home the same day following recovery from the anaesthetic.

### 2.2. Dietary and Physical Activity Counselling

All participants’ dietary history and current dietary behaviour were assessed at baseline. A qualified dietitian counselled participants regarding their diet and physical activity. The dietary counselling programme was intended to provide each participant with lifestyle and behavioural modification information and impart good eating practices. Guidelines for daily total requirements were between 1200 and 1500 kilocalories for women and between 1500 and 1800 kilocalories for men. Participants were advised to eat regularly every day (five times/day), to control their portion sizes, to increase their intake of low glycaemic index (GI) and high-protein foods, and to reduce their intake of foods high in fat, sugar, and alcohol.

Participants in both groups were advised to include more physical activity in their daily routine, like walking more every day and climbing the stairs instead of taking the lift or escalators. They were also asked to start with short periods of low-intensity exercise and increase the intensity and duration slowly. Their goal was to include 150 min/week of moderate-intensity and 75 min/week of vigorous-intensity aerobic activity and muscle-strengthening activities more than two days a week.

### 2.3. Liquid Diet

All participants followed a liquid diet during the seven days before and 13 days (±3 days) after the DJBL insertion, or the fourth clinical visit for the control group. The liquid diet was based on a liquid meal replacement—Fortisip compact^®^ meal replacements (Nutricia Ltd., Trowbridge, UK): four bottles (125 mL each, energy: 300 kcal; carbohydrates: 49%; fat: 35%; protein: 16%) for women and five bottles for men daily. Allowed in addition to this were: milk, flavoured milk, water, low-sugar squashes, vegetable juices, tea or coffee without sugar, unsweetened puree fruit juice, or clear soups. After the liquid diet, participants in both groups were advised to follow a low-calorie diet.

### 2.4. Anthropometric Measurements

Weight was measured at all visits, in bare feet, and wearing light clothes. Height without shoes was recorded at the baseline visit. Body mass index (BMI) was calculated. Percentage of body composition (fat mass, fat-free mass in kg and %) were obtained using a bio-electrical impedance analysis machine MC-780MA (TANITA Corporation, Japan).

### 2.5. Food Intake and Macronutrient Selection

Participants were asked to complete a weighed food diary for 3 days, 2 weekdays and 1 weekend at baseline (2 weeks before intervention), 10 days, 6 months, 12 months, and 24 months post-intervention. Information from the diaries was entered and analysed using Dietplan7 software (Forestfield Software Ltd. West Sussex, UK) to obtain total daily caloric intake and percentage contribution from carbohydrates, protein, and fat.

### 2.6. Assessment of Taste Function

#### 2.6.1. Sensory Domain of Sweet Taste

The detection threshold for sweet taste was measured using the method of constant stimuli [9]. In brief, 112 polystyrene cups were presented in 8 blocks; each block consists of 14 cups, including 7 concentrations of sucrose and 7 water stimuli randomly organised. An amount of 15 mL of the sucrose solution and water was presented in each cup. Participants were asked to taste the solution, swirl it around properly and expel it without swallowing. Then they were asked to describe the quality of the solution they were testing, if it was sweet or water. After each stimulus participants were asked to rinse their mouth with water (the same water used to prepare the solutions) before tasting the next stimulus. Participant answers were recorded on a template scoring sheet. The detection test was performed on the morning of the study day after an overnight fast. All solutions were prepared using water (Caledonian Still Natural Mineral Water, Sainsbury’s Supermarkets Ltd., London, UK) and sucrose (Sigma-Aldrich, Dorset, UK) and presented at room temperature. Seven sucrose concentrations were used (2.1, 6.25, 12.5, 25, 50, 100, and 300 mM). All participants performed the above-described test at baseline (2 weeks before intervention), at 10 days, and at 6 months. Participants were asked to come to the research facility at 8 am after over-night fast. Each visit had a different random arrangement of the cups to minimise learning and familiarisation.

The data collected from the sucrose detection test allows for the derivation of a psychometric function, which is a mathematical equation that plots the performance of participants against the physical aspect (concentration) of the stimulus. The performance was measured as a percentage of correct responses (responses where the participant was able to detect the stimulus correctly).

A ‘hit’ was defined as when the participant correctly reported that the stimulus was different from water when sucrose was presented. A ‘false alarm’ (FA) was defined when the participant incorrectly reported that the stimulus was different from water when water was presented. The hit rate for a given sucrose concentration was adjusted for the false alarm rate to derive a ‘corrected hit rate’ using the following equation:

$$Corrected\;hit\;rate\;=\;\frac{P\left(hit\right)\;-\;P\left(FA\right)}{1.0\;-\;P\left(FA\right)}$$

where *P*(*hit*) = the proportion of sucrose trials (cups) of a given concentration that were hit, and *P*(*FA*) = the proportion of water trials that were false alarms. Thus, when the uncorrected hit rate is equal to the false alarm rate, the corrected hit rate = 0.

Concentration–response curves were fitted to the corrected hit rate values for each participant for the three tested occasions (2 weeks pre, 10-days and 6 months post-intervention) to derive a family of individual psychometric functions using the following logistic equation:

$$f\left(x\right)\;=\;\frac{a}{{1\;+\;10}^{\left(\left(\log10\left(x\right)\;-\;c\right)*b\right)}}$$

where log10(x) = log10 concentration, a = the upper asymptote of performance (maximum performance = 1), b = slope, and c = the log10 concentration at ½ a performance (i.e., EC50, defined as half-maximal effective concentration). We defined the c parameter as the threshold because it represents the inflexion point of the psychometric function and thus optimally represents horizontal shifts in the sensitivity.

Only c-values of the individual curve fits for the participants who had fits that accounted for at least 85% of the variance were compared. C-values were calculated using Mystat^®^ (Systat^®^ 12) software (Cranes Software International Ltd., Palo Alto, CA, USA). The shifts in the c parameters between groups and within groups were assessed.

#### 2.6.2. Appetitive Reward Domain of Taste Function

The appetitive reward value of sweet/fat taste was measured using the validated method of the progressive ratio task [10]. In brief, this is a computer task in which participants were seated in front of a screen with a plate of 20 chocolate candies (M&M^®^ crispy candies, Mars UK Limited, Slough, UK), each one containing approximately 4 kcal (energy contribution: 43.7% sugars, 44.1% fat). They were asked to click on the mouse button continuously until they received a message on the screen, allowing them to consume their reward (one M&M’s only). The required number of clicks increased progressively after each reward (candy). The first ratio was ten clicks with a geometric increase of two (i.e., 10, 20, 40, 80, etc.) for every ratio afterwards. Participants were allowed to terminate the task at any point by pressing the spacebar button on the keyboard. This test was carried out on two occasions, two weeks pre- and six months post-intervention. Testing occurred 3 h after consuming a standardised meal of 250 mL of Fortisip Compacts vanilla flavour, (Energy: 600 kcal, carbohydrates: 74.2 g, fat: 23.2 g, protein: 24 g). The total number of clicks and clicks in the last completed ratio (breakpoint) were recorded. In addition, the number of consumed and remaining candies were calculated from the plate after the termination of the task to cross-check and validate the participants followed the instructions. Comparisons between groups and within groups were assessed.

#### 2.6.3. Consummatory Reward Domain of Taste Function

The consummatory reward value of sweet taste was measured using a validated methodology [9]. In brief, 30 polystyrene cups were presented in 3 blocks; each block consists of 10 cups, comprising 5 cups of the 5 different sucrose concentrations and 5 cups of water for rinsing after sweet solutions. Odd number cups contained the sucrose solutions, and even number cups contained the rinsing water. An amount of 15 mL of the sucrose solution and water was presented in each cup. All solutions were stored at 4 °C and presented cold for testing. Solutions were prepared using still water (Caledonian Still Natural Mineral Water, Sainsbury’s Supermarkets Ltd., London, UK: pH 7.4, calcium 27 mg/L, chloride 6.4 mg/L, bicarbonate 103 mg/L, magnesium 6.9 mg/L, sulphate 10.6 mg/L, sodium 6.6 mg/L). Sucrose was from (Sigma-Aldrich, Dorset, UK), five different concentrations of sucrose were used (0, 50, 100, 200, 400 mM). Two different visual analogue scales were used to assess the liking of the sweet drinks as follows:

The Hedonic General Labeled Magnitude Scale: This visual analogue scale was used to rate the pleasantness of the sweetness of the solution relative to any liking feeling they had ever experienced. This is a vertical scale with the middle anchor representing the ideal rating (‘Neutral’) with a value of zero (0), and measurements of the most positive (‘Strongest liking of any kind’) representing the highest value of +100, and most negative rating (‘Strongest disliking of any kind’) with the least value of −100 located at the lowest end of the scale.

The ‘Just About Right’ scale: This visual analogue scale was used to compare the sweetness of the solution as compared to the ideal sweetness of the participant’s preferred soft drink. This was a vertical visual analogue scale, having a middle point where the ideal rating was situated *(‘Just right: My ideal sweetness in a drink’)* which corresponded to the value of zero (0), while the upper end of the scale measured the most positive *(‘Far too sweet: I would never drink it)* corresponded to a value of +100, and the most negative rating *(‘Far too little sweetness: I would never drink it’)* which corresponded to a value of −100, and this was at the lower end of the scale.

All participants performed the above-described test on three occasions: 2 weeks pre-intervention, 10 days, and 6 months post-intervention. Participants completed this test after the sensory domain task and still in the fasting state. Each visit had a different random arrangement of the cups to minimise learning and familiarisation.

### 2.7. Statistical Analyses

The mixed model analysis was used to investigate the treatment effect on the variables of interest over time, allowing us to perform both between-groups and within-group comparisons. The model included fixed effects for the visit (time of assessment), group (DJBL or control) and their corresponding interaction (group×visit), as well as a random intercept effect for each patient. The model was adapted to include a third level where appropriate (for example, sucrose concentrations).

All participants who attended baseline and at least one visit were included in the analysis. Analysis results are presented in the form of Type-III test results of fixed effects (*p*-values) and their subsequent estimates (mean ± SD). Any parameter that produced a significant result (*p* < 0.05) in the analysis was considered for post-hoc testing of least-square means to investigate any potential effect in more detail. The Pearson test was used for linear regressions. Statistical analysis was completed using IBM statistics SPSS 24, and graphs were generated using GraphPad Prism version 8.

The trial was approved by the Fulham Research Ethics Committee on 10 July 2014 (reference 14/LO/0871) and conducted in accordance with the Declaration of Helsinki.

## 3. Results

### 3.1. Baseline Characteristics

Forty-seven participants took part in this study, 27 in the Endobarrier group and 20 in the control group, 55% of the participants were male (Table 1). Within the control group, 1 participant withdrew from the study at visit 5 (10 days after intervention), 4 participants withdrew at visit 8 (6 months after intervention). Within the Endobarrier group, 2 participants withdrew from the study at visit 5 (10 days after intervention), 5 participants withdrew at visit 8 (6 months after intervention).

### 3.2. Body Weight

**Figure 2:**
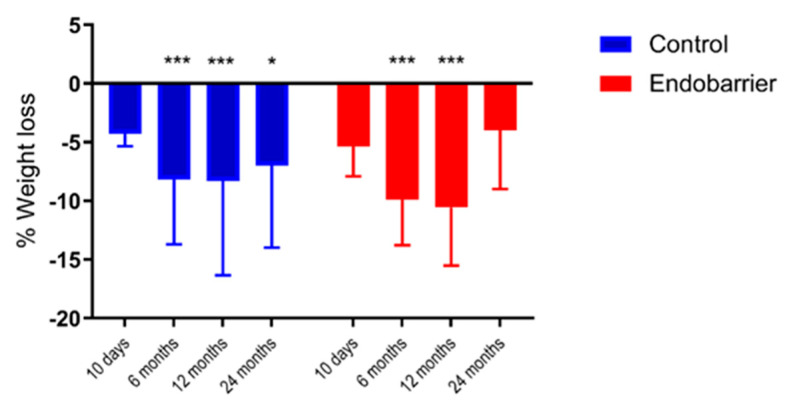
Percentage weight loss throughout the study. * *p* < 0.05, *** *p* < 0.001 compared to baseline within the same group. Data given as mean ± SD.

There was a significant reduction in total body weight within each group at 10 days, 6 and 12 months compared to baseline (*p* < 0.001) but no significant differences between groups. At 12 months the Endobarrier group lost 11 ± 5% total body weight vs. 8 ± 8% in the control group, while at 24 months, the Endobarrier group lost 4 ± 5% vs. 7 ± 7% in the control group ((*p* < 0.02, Figure 2).

### 3.3. Food (Energy) Intake and Macronutrient Selection

Total daily caloric intake from the three-day food diary was significantly reduced within both groups at all time points compared to baseline, but there were no significant differences between the groups (Table 2).

Within the Endobarrier group, there was a significant increase in the % contribution from carbohydrates at 10 days, a significant increase in the % contribution from protein at 12 months, and a significant decrease in the % contribution from fat at 10 days. Within the control group, there was a significant increase in the % contribution from carbohydrates at 10 days, a significant decrease in the % contribution from protein at 10 days and an increase at 6 months. However, there were no significant differences between groups (Table 3).

### 3.4. Sensory Domain of Sweet Taste

**Figure 3:**
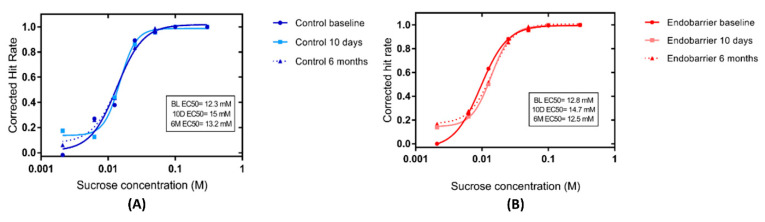
Sweet taste detection. Curves of the mean corrected hit rate over time for (**A**) controls (blue) *n* = 16 and (B) Endobarrier (red) *n* = 25 groups as a function of sucrose concentration. The EC50 was derived from the c-parameter in the curve fit and represented the concentration at which the corrected hit rate reaches 50% of the maximum asymptote.

There was no significant change in the curves of mean corrected hit rate both within and between groups at baseline, 10 days, and 6 months post intervention (Figure 3).

### 3.5. Appetitive Reward Value of Sweet Taste

**Figure 4:**
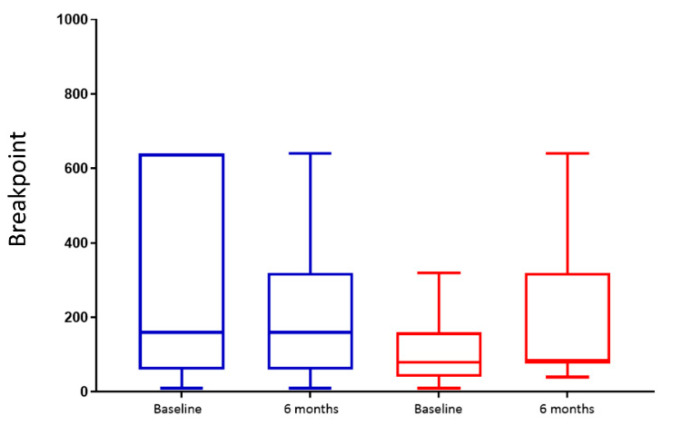
Breakpoint at the progressive ratio task. Box plot of the breakpoint for chocolate candies in control (blue) *n* = 9 and Endobarrier (red) *n* = 11 groups. The lower and upper boundaries of the box represent 25th and 75th percentiles, respectively. Lower and upper whiskers represent 10th and 90th percentiles, respectively. The line in the middle of the box represents the median.

There was no significant change in the breakpoint either within or between groups (*p* = 0.12 for group × time interaction) (Figure 4).

### 3.6. Consummatory Reward Value of Sweet Taste

**Figure 5:**
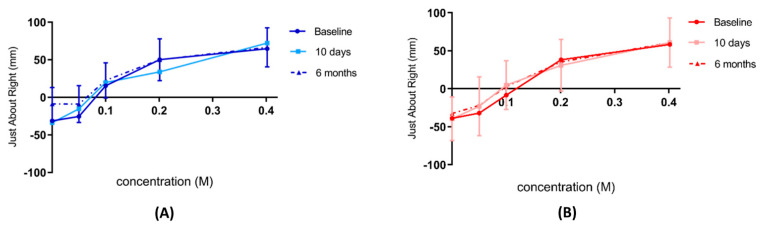
Just About Right scale ratings of sweet taste. Consummatory reward value of sweet taste assessed by Just About Right scale for (**A**) controls *n* = 19 (blue) and (**B**) Endobarrier *n* = 24 (red groups). Data are presented as the mean rating at each concentration ± SD.

**Figure 6:**
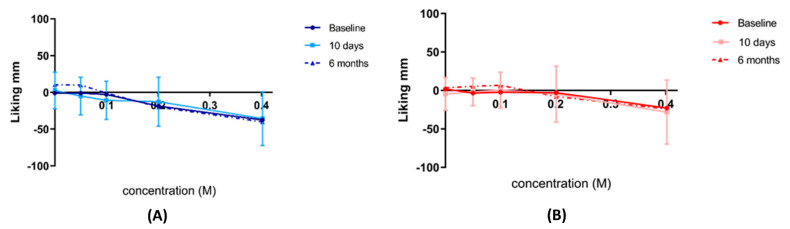
Hedonic general Labeled Magnitude Scale ratings of sweet taste. Consummatory reward value of sweet taste assessed by the Hedonic general Labeled Magnitude Scale for (**A**) control *n* = 19 (blue) and (**B**) Endobarrier *n* = 24 (red) groups. Data are presented as the mean rating at each concentration ± SD.

There was no significant change in the consummatory reward value of sweet taste both within and between groups using Just About Right scale (Figure 5) and Hedonic General Labeled Magnitude Scale (Figure 6) at baseline, 10 days, and 6 months post intervention.

## 4. Discussion

This is, to our knowledge, the first experimental medicine study to assess the mechanisms of action of the Endobarrier device on weight loss as a nested study within an RCT. Patients in the Endobarrier group lost numerically more weight than the control group. We assessed several measures of dietary behaviour and identified significant changes on specific aspects within groups but no significant differences between groups.

To date, there has been limited literature on the effect of Endobarrier on food intake. Using food diaries, our results demonstrated similarly reduced food intake within both the Endobarrier and the control groups. Similarly, a recent case series study of patients with obesity and T2DM demonstrated reduced food intake at 36 weeks after the Endobarrier implant using a semi-quantitative Food Frequency Questionnaire [11]. In contrast, a prospective observational study of two groups, a group of patients with obesity and normal glucose-tolerance, and another group of matched metformin-treated patients T2DM who underwent Endobarrier implant, demonstrated lower food intake only at one week [12]. This was followed by a return to baseline food intake at explantation (26 weeks), despite ongoing weight loss. This was the only human study so far to use an ad libitum meal to assess food intake. Of note, this study also did not include a control group for comparison [12]. Among animal models, a study comparing food intake between diet-induced obese rats after endoluminal sleeve insertion and sham-operated controls showed reduced food intake in the sleeve group compared to no change in the control group at eight weeks [13].

Alternative mechanisms of weight loss after Endobarrier have been proposed including increased energy expenditure in both human and animal models. We cannot exclude that the numerically superior weight loss observed among our Endobarrier group might be attributed to an increase in energy expenditure. Rohde et al. reported an increase in resting energy expenditure using indirect calorimetry in patients with obesity but not among patients with T2DM after Endobarrier implant [12]. Similarly, Munoz et al. in their animal model, demonstrated an increase of 13% in total and 9% in resting energy expenditure among Endoluminal sleeve treated rats compared to shams [13].

Nutrient malabsorption has been proposed as a possible mechanism of weight loss after the Endobarrier, due to the bypass of 60 cm of the small intestine. However, when fat malabsorption was measured using ^13^C mixed triglyceride breath test in patients with obesity and T2DM, there was no evidence of reduced intraluminal lipolytic activity suggesting that fat malabsorption does not take place [14]. Similarly, no evidence of food malabsorption was found in rats treated with an endoluminal sleeve, as measured by the difference in calories consumed and excreted in the stool using direct calorimetry [13].

Another plausible mechanism that could explain the weight loss in the Endobarrier group is gut inflammation. The insertion of a foreign body in the intestine could have triggered a low-grade inflammatory state. Gut inflammation can cause weight loss due to several mechanisms including increased resting energy expenditure, and the action of proinflammatory cytokines [15]. Against this hypothesis is the fact that we measured plasma concentrations of C-reactive protein in the main clinical RCT [6] and were not found to be elevated after intervention. We would also have expected gut inflammation to decrease appetite and thus total daily energy intake, but we did not observe this in our study. In this report we do not present appetite ratings or gut hormone measurements. Whilst enhanced post-prandial concentrations of plasma GLP-1 and PYY were reported in some studies [16,17,18], the magnitude of the increase was modest and the findings inconsistent [16,19].

Weight regain after Endobarrier explant is reported in several studies. Interestingly, in the studies that had a control group, the Endobarrier group had the most weight regain compared to the control group [20]. This was in line with our findings, where the Endobarrier group had around 7% weight regain compared to 1% in the control group. Similarly, Villarasa et al., in their recent prospective trial concluded a total percentage weight loss of about 15% at the time of explantation (48 weeks) followed by weight regain during the next year, maintaining only 7% of the total weight loss; there was no control group in this study [17].This rebound demonstrates that the Endobarrier works only when it is in situ and does not have any long term learning effects on eating behaviour. One explanation for the magnitude of the rebound might be attributed to the absence of abdominal discomfort that patients commonly report, resulting in increased meal size, caloric intake and subsequent weight regain.

The role of the duodenum in food preferences and reward has been investigated in animal models of the DJB procedure, which like the Endobarrier, involves bypass of the proximal small intestine [5]. In line with our results, Qu et al. recently demonstrated that sweet preference was not different between DJB mice and sham-operated mice in a two-bottle sweet preference test [21]. Reduced preference appeared only after prolonged exposure to the sweet solutions indicating a learning effect [21]. Similarly, in their animal model, Zhang et al. demonstrated that DJB mice preferred the flavours of intragastric infusions of metabolised glucose compared to the flavours of non-metabolised glucose [22]. Nevertheless, the surgical duodenal bypass did not affect the ability of mice to differentiate (prefer) between the flavours of metabolised versus non-metabolised glucose solutions [22]. The same study also showed that reward circuits in the brain responded to intra-portal mesenteric infusions of the metabolised glucose only, suggesting a post-absorptive role for glucose preference and reward.

The absence of changes in food preference and taste function are reminiscent of some of the studies in humans and animals undergoing RYGB [23,24]. In one of the most comprehensive studies in the literature, food preferences did not change in a group of patients undergoing RYGB, but the subgroup of patients who experiences changes in food preferences lost more weight [2]. This finding suggests that changes in food preferences do not take place in everyone but in those that do, they contribute to weight loss as an additional mechanism.

The strengths of this study include its randomised design, two trial sites, length of follow-up, multidisciplinary team involved in patients care, and delivery of an intensive medical intervention throughout the study period. In addition, we used complementary measures of eating behaviour, including assessment of food intake, taste detection thresholds, appetitive and consummatory reward value of sweet taste with various sweet concentrations. Despite the length of the study, the same two dietitians carried out dietary analyses throughout the study period to reduce variability. In addition, participants in both groups received the exact behavioural and dietary modification instructions from a single dietitian throughout the study.

The major limitation in our study was its unblinded design. In addition, in the smaller mechanistic sub-set of participants having these dietary assessments, the Endobarrier insertion resulted in numerically superior weight loss, which was not as pronounced as in the main RCT [6]. There are also inherent limitations to using verbal/written reports, especially in a trial that is not double-blinded. The problem of under-reporting of food intake among patients with and without obesity is common when using indirect measures of food intake [25]. It would have been preferable to measure these aspects of eating behaviour using a buffet meal or a 24-h residential stay. The study days were long and included numerous tasks which might have contributed to participant fatigue, which could have been avoided if the tasks had been performed on separate days. Only sweet taste assessments were made and not fat or combined sweet/fat (other than the progressive ratio task). Assessments were generally performed in the fasted state and may have been different in the post-prandial state, except for the progressive ratio task, which was assessed post prandially Furthermore, sample sizes declined over time due to drop-out during the trial. Finally, we did not measure energy expenditure or calorie malabsorption as alternative mechanisms causing weight loss after the Endobarrier.

## 5. Conclusions

In conclusion, this experimental medicine study demonstrated that reduction of self-reported energy intake, changes in food preferences, and sweet taste were not the mechanisms underlying the weight loss observed after Endobarrier insertion in people with obesity and T2DM.

## Figures and Tables

**Table 1:** Baseline characteristics of participants.

	Control (*n* = 20)	Endobarrier (*n* = 27)
Age (years)	54 ± 6	52 ± 8
Sex (M/F)	8/12	18/9
Weight (kg)	101.3 ± 14.4	109.4 ± 18.9
BMI (kg/m^2^)	36 ± 4	36 ± 5
Bio-impedance body fat (%)	42 ± 8	39 ± 7
HbA1c (mmol/mol)	70 ± 12	76 ± 11
Diabetes duration (years)	7 (1–25)	8 (2–19)
HOMA-IR	5.43 ± 3.6	5.36 ± 1.8

Data are presented as mean ± SD or median (range).

**Table 2:** Total daily caloric intake (kcal).

	Group								Mixed Model Analysis	
	Control				Endobarrier				Effect	*p*-Value
	*n*				*n*					
Baseline	17	1740	±	285	24	1911	±	506		
10 Days	17	1194	±	203 ***	22	1097	±	407 ***	Group	0.51
6 months	14	1443	±	321 *	16	1575	±	410 **	Time	<0.001
12 months	13	1504	±	470 *	13	1423	±	647 ***	Group × Time	0.25
24 months	14	1525	±	494	12	1788	±	761		

Results presented as mean ± SD. * *p* < 0.05 ** *p* < 0.01 *** *p* < 0.001 compared to baseline within the same group.

**Table 3:** Percentage contribution of macronutrient to daily energy intake.

	Group								Mixed Model Analysis	
	Control				Endobarrier				Effect	*p*-Value
Carbohydrates (% of Total Caloric Intake)										
	*n*									
Baseline	17	40	±	8	24	40	±	7		
10 Days	17	47	±	2 **	22	46	±	6 **	Group	0.83
6 months	14	39	±	9	16	41	±	7	Time	<0.001
12 months	13	41	±	9	13	37	±	8	Group × Time	0.50
24 months	14	40	±	7	12	42	±	7		
**Protein (% of Total Caloric Intake)**										
Baseline	17	19	±	4	24	19	±	5		
10 Days	17	16	±	1 *	22	19	±	6	Group	0.89
6 months	14	24	±	5 **	16	21	±	6	Time	<0.001
12 months	13	21	±	4	13	22	±	7 *	Group × Time	0.05
24 months	14	22	±	7	12	19	±	5		
**Fat (% of Total Caloric Intake)**										
Baseline	17	38	±	7	24	38	±	6		
10 Days	17	37	±	2	22	35	±	4 *	Group	0.90
6 months	14	36	±	10	16	36	±	7	Time	0.50
12 months	13	36	±	9	13	38	±	7	Group × Time	0.60
24 months	14	37	±	8	12	36	±	7		

Results presented as mean ± SD. * *p* < 0.05 compared to baseline within the same group. ** *p* < 0.01 compared to baseline within the same group.

## Data Availability

The data presented in this study are available on request from the corresponding author. The data are not publicly available due to privacy.
